# Go Slow in New York State

**Published:** 1897-03

**Authors:** 


### GO SLOW IN NEW YORK STATE.

THE proposed measure to create the office of State Veterinarian in the Empire State and to associate the same with the State Board of Health is a radical one, which needs the careful, earnest consideration of the entire veterinary profession before it is pushed to its passage. For many years the work of such an officer has been associated in nation and State, at home and abroad, with departments of agriculture, in whose interests primarily such officials were created; and it is well to consider whether it is wise, whether the time and occasion are upon us when there are sufficiently good reasons for its divorcement therefrom. The agricultural and animal industries have had more than their share of the burden in the past few years. Needless and fruitless agitation has gone on to further imperil their security and prosperity, and this at times has been unmindful of just and adequate protection and recompense for their losses and burdens, and to no one must they look for aid and assistance as much as to the veterinarian. To render assistance to them, and through them to the public, we must seek to obtain their confidence and co-operation, or our efforts will be only half successful and the results disappointing to all concerned. There are reasons why the association of this office with a State Board of Health would give it added power and usefulness; but do these outweigh the dangers that are likely to follow the widening of the gap between the producer and consumer of animal food-products?

